# A *BCR-ABL1* cutoff of 1.5% at 3 months, determined by the GeneXpert system, predicts an optimal response in patients with chronic myeloid leukemia

**DOI:** 10.1371/journal.pone.0173532

**Published:** 2017-03-09

**Authors:** Valentín García-Gutiérrez, María T. Gómez-Casares, José M. Puerta, Juan M. Alonso-Domínguez, Santiago Osorio, Juan C. Hernández-Boluda, Rosa Collado, María J. Ramírez, Fátima Ibáñez, María L. Martín, Juan D. Rodríguez-Gambarte, Carolina Martínez-Laperche, Montse Gómez, Dolly V. Fiallo, Sara Redondo, Alicia Rodríguez, Concepción Ruiz-Nuño, Juan L. Steegmann, Antonio Jiménez-Velasco, Spanish Group of Chronic Myeloid Leukemia (GELMC)

**Affiliations:** 1 Hematology Department, Hospital Universitario Ramón y Cajal, Madrid, Spain; 2 Hematology Department, Hospital Universitario Doctor Negrín, Las Palmas de Gran Canaria, Spain; 3 Unidad de Gestión Clínica Hematología y Hemoterapia, Hospital Universitario Virgen de las Nieves, Granada, Spain; 4 Hospital Universitario Fundación Jiménez Díaz, Madrid, Spain; 5 Hematology Department, Hospital Universitario Gregorio Marañón, Madrid, Spain; 6 Hematology Department, Hospital Clínico, Valencia, Spain; 7 Genetic Laboratory, Hematology Department, Consorcio Hospital General Universitario, Valencia, Spain; 8 Hematology Department, Hospital de Jerez de la Frontera, Cádiz, Spain; 9 Hematology Department, Hospital San Pedro de Alcántara, Cáceres, Spain; 10 Hematology Department, Hospital Universitario Virgen Macarena, Sevilla, Spain; 11 Hematology Department, Hospital Regional Universitario de Málaga, IBIMA, Málaga, Spain; 12 Hematology Department & IIS-IP, Hospital Universitario de la Princesa, Madrid, Spain; University of Thessaly Faculty of Medicine, GREECE

## Abstract

In chronic myeloid leukemia (CML) patients, 3-month *BCR-ABL1* levels have consistently been correlated with further outcomes. Monitoring molecular responses in CML using the GeneXpert (Cepheid) platform has shown an optimal correlation with standardized RQ-PCR (IS) when measuring *BCR-ABL1* levels lower than 10%, as it is not accurate for values over 10%. The aim of the present study was to determine the predictive molecular value at three months on different outcome variables using the Xpert *BCR-ABL1* Monitor^TM^ assay (Xpert *BCR-ABL1*). We monitored 125 newly diagnosed consecutive CML patients in the chronic phase (CML-CP) using an automated method: Xpert *BCR-ABL1*. Only 5% of patients did not achieve an optimal response at 3 months, and the 10% *BCR-ABL1* cutoff defined by RQ-PCR (IS) methods was unable to identify significant differences in the probabilities of achieving a complete cytogenetic response (CCyR) (50% vs. 87%, p = 0.1) or a major molecular response (MMR) (60% vs. 80%, p = 0.29) by 12 months. In contrast, a cutoff of 1.5% more accurately identified differences in the probabilities of achieving CCyR (98% vs. 54%, p<0.001) and MMR (88% vs. 56%, p<0.001) by 12 months, as well as probabilities of treatment changes (p = 0.005). Therefore, when using the Xpert *BCR-ABL1* assay, a cutoff of 1.5% at 3 months could with high probability identify patients able to achieve an optimal response at 12 months.

## Introduction

Over the last decade, with the incorporation of tyrosine kinase inhibitors (TKIs) for the treatment of chronic myeloid leukemia (CML), the expected overall survival of these patients has reached that of the general population at the same age interval [1]. However, some patients do not respond favorably to TKIs, and the probabilities of progression and death remain high in this patient subset [2]. Thus, the early identification of this group of patients is crucial for determining treatment changes [3]. Real-time quantitative PCR (RQ-PCR) is the molecular procedure recommended by a panel of experts for monitoring CML patients [4]. Nevertheless, conventional RQ-PCR analysis of *BCR-ABL1* transcripts is a labor-intensive procedure, incorporating numerous pre- and post-analytical phases, all of which are potential sources of variation and require rigorous technical attention [5]. In recent years, there has been increased effort concerning the standardization of molecular monitoring, and the International Scale (IS) for *BCR-ABL1* RQ-PCR quantification has been widely adopted to mitigate the effects of variability between laboratories.

Unfortunately, these initiatives do not address all aspects of the testing process, leading to variable results [6]. Thus, new automated procedures have been incorporated to simplify the process and time spent on molecular monitoring. The Xpert *BCR-ABL1* PCR system, performed using a GeneXpert automated analyzer, is a cartridge-based assay with minimal pre-analytical and analytical states enabling faster and easier *BCR-ABL1* monitoring than RQ-PCR [7]. Our previous studies, as well as those of other groups, have shown a good correlation between Xpert *BCR-ABL1* and conventional RQ-PCR for values under 10% [8–10]. Similar results were observed by Enjeti et al. [11] and O’Dwyer et al. [12] in prospective studies comparing these two methods.

A cutoff of 10% at 3 months using conventional RQ-PCR (IS) has consistently been correlated with clinically relevant outcomes, including the probabilities of achieving a complete cytogenetic response (CCyR), major molecular response (MMR), progression-free survival (PFS) and OS, in patients treated with frontline imatinib and a second-generation TKI (2GTKI) [13–18]. Thus, NCCN and ESMO guidelines have recommended changes for the treatment of patients who do not achieve this milestone at 3 months [3,19]. Conversely, ELN recommendations do not consider this group of patients as failures but as patients that should be closely monitored, reflecting the high risk of subsequently fulfilling failure criteria during follow-up [20].

The aim of the present study was to validate the predictive value on different outcome variables of molecular responses at 3 months using the Xpert *BCR-ABL1* assay, including outcomes defined as “optimal” by ELN recommendations such as achieving MMR and CCyR at 12 months [20].

## Methods

### Patients and methods

We examined 125 new consecutive CML chronic phase (CML-CP) patients diagnosed and followed up in 13 Spanish centers between 2010 and 2013. All patients were Philadelphia chromosome-positive with confirmed *BCR-ABL1* rearrangement (e13a2 or e14a2 transcripts) based on qualitative PCR at presentation. The baseline characteristics of the patients are shown in Table 1. The present study was approved by the Hospital Universitario Ramón y Cajal Research ethics committee. All clinic hematologic data from each patient was collected after written inform consent, approved by the ethic committee, was obtained. Signed informed consents were recorded within patient's clinical history.

**Table 1:** Baseline characteristics.

Sex (male/female)	75/50
**Age (years)**	
Median	51
Q1	44
Q3	67
**Hemoglobin level (gr/dl)**	
Median	12.7
Q1	11.55
Q3	14.05
**Platelet count (x 10**^**9**^**/L)**	
Median	496
Q1	259
Q3	562
**Basophils in peripheral blood (%)**	
Median	3
Q1	0.7
Q3	5.8
**Blasts in peripheral blood (%)**	
Median	0.9
Q1	0
Q3	1.2
**Median spleen size below the costal margin (cm)**	
Median	3
Q1	0
Q3	10
**Sokal score, n (%)**	
Low risk	53 (43)
Intermediate risk	50 (40)
High risk	21 (17)
Missing	1
**Hasford score, n (%)**	
Low risk	60 (50)
Intermediate risk	56 (47)
High risk	4 (3)
Missing	5
**Eutos score, n (%)**	
Low risk	100 (82)
High risk	21 (18)
Missing	4

The monitoring and treatment strategies were selected at the discretion of the hematologist, thereby reflecting the actual treatment of CML patients in clinical practice. The first-line treatments consisted of imatinib (IM), nilotinib (NI), dasatinib (DA) and bosutinib (BO) in 58% (73), 28% (34), 13% (17), and 1% (1) of the patients, respectively.

*BCR-ABL1* transcript quantification was performed using the automated method Xpert *BCR-ABL1* Monitor™, Cepheid, aligned to the 0.1% *BCR-ABL1* ratio according to the standards of the World Health Organization [7]. The samples were analyzed according to the manufacturer’s recommendations.

### Evaluation of the responses

Cytogenetic responses were defined according to ELN definitions. Particularly, CCyR was defined as the absence of detectable Philadelphia chromosome in 20 mitotic cells and/or ≤1% *BCR-ABL1* cells based on the interphase FISH analysis of 200 nucleated cells.

A molecular response was described as MMR when *BCR-ABL1*/*ABL1* transcript levels were ≤0.1% (IS), whereas MR4.0 was defined as either detectable disease ≤0.01% *BCR-ABL1* (IS) or *undetectable BCR-ABL1* with a detection limit of 0.01%. MR4.5 was defined as either detectable disease ≤0.0032% *BCR-ABL1* (IS) or undetectable *BCR-ABL1* with a detection limit of 0.0032%, and MR5.0 was defined as either detectable disease ≤0.001% *BCR-ABL1* (IS) or undetectable *BCR-ABL1* with a detection limit of 0.001%.

An event was defined as the loss of CCyR or complete hematological response (CHR), progression to accelerated or blastic phase, need for treatment change or death for any reason.

### Statistical methods

Data on continuous variables are presented as medians and quartiles. Categorical variables were analyzed based on absolute and relative frequencies.

The proportions of patients who achieved MMR and CCyR after first-line treatment for 1 year and the response at 3 months were compared by applying Pearson’s chi-square test or Fisher’s exact test, depending on the expected frequency counts of the cells. All analyses were performed on an intention-to-treat basis unless otherwise stated. A p-value <0.05 was considered significant, and the contrasts were bilateral.

To determine the best cutoff for the *BCR-ABL1*/*ABL1* level using Xpert *BCR-ABL1* at 3 months to predict CCyR and MMR at one year, a receiver-operating characteristic (ROC) curve was generated. The same methodology was followed as previously described but applying the newly obtained threshold.

To identify potential covariates, multivariate analyses were performed using logistic regression to predict CCyR and MMR after 1 year, introducing the classification of the patients according to the new threshold at 3 months together with age, gender and Sokal score.

## Results

### Outcomes and responses

The median follow-up for the entire cohort was 43 months (range: 14–61). The overall probability of achieving CCyR and MMR at 12 months was 84% (106/125) and 68% (86/125), respectively. Table 2 shows the CCyR and MMR probabilities for each specific TKI used in first-line therapy. Twenty-nine patients (23%) required treatment changes, reflecting resistance (n = 12) or intolerance (n = 17). Among all patients receiving frontline treatment with imatinib, 23 of 73 patients received 2GTKI (dasatinib or nilotinib) as second-line treatment. Six of the 52 patients treated with 2GTKI (nilotinib, dasatinib or bosutinib) frontline therapy required treatment changes: 3 patients were changed to imatinib (2 patients showed intolerance and 1 patients showed primary resistance), 1 patient received ponatinib (reflecting primary resistance), and 1 patient received stem cell transplantation. Mutational analysis was performed in patients with failure criteria by ELN recommendations at any time point, nevertheless it was not performed in neither of the six patients who did not achieve a *BCR-ABL1* levels below 10% at 3 months. Twenty-nine percent (37/125) of the patients suffered an event during follow-up, but only 2 patients progressed to blast crisis (1.6%). Eight (6.4%) patients died (6 patients died of causes not related to CML) during the follow-up period. The two patients who progressed to blast crisis died, both patients were treated with imatinib first line and none of them achieved an optimal response by 3 months. All of the six patients who died of causes not related to CML achieved an optimal response by 6 months.

**Table 2:** Complete cytogenetic responses and major molecular response probabilities for each specific TKI used in first-line therapy.

12-month responses	Imatinib	Nilotinib	Dasatinib	Bosutinib	Total
	(n = 73)	(n = 34)	(n = 17)	(n = 1)	(n = 125)
**CCyR**					
**Yes**	78%^a^	91%^b^	100%^c^	100%^d^	84%^e^
	(57/73)	(31/34)	(17/17)	(17/17)	(106/125)
**No**	22%	9%	0%	0%	16%
	(16/73)	(3/34)	(0/17)	(1/1)	(19/125)
**MMR**					
**Yes**	63%^f^	84%^g^	64%^h^	100%^i^	68%^j^
	(46/73)	(28/34)	(11/17)	(1/1)	(86/125)
**No**	37%	16%	36%	0%	32%
	(27/73)	(6/34)	(9/17)	(0/1)	(39/125)

^a^ 72/73 patients (98%) treated with imatinib were evaluated.

^b^ 31/34 patients (93%) treated with nilotinib were evaluated.

^c^ 100% of patients treated with dasatinib were evaluated.

^d^ 100% of patients treated with bosutinib were evaluated.

^e^In total, 122/125 (97%) patients were evaluated.

^f^62/73 (84%) patients treated with imatinib were evaluated.

^g^100% of patients treated with nilotinib were evaluated.

^h^12/17 (70%) of patients treated with dasatinib were evaluated.

^i^100% of patients treated with bosutinib were evaluated.

^j^In total, 109/125 (87%) patients were evaluated.

### Three-month response and its correlation with subsequent outcomes

At 3 months, molecular assessments were performed in 112 patients. No patients died or progressed during the first 3 months of follow-up.

We attempted to validate the 10% cutoff using the Xpert *BCR-ABL1* method as a surrogate endpoint to predict subsequent outcomes. Only 6/112 (5%) patients had *BCR-ABL1* levels >10% at 3 months, a value significantly lower than historical series when using standardized RQ-PCR. All patients treated with 2GTKI achieved *BCR-ABL1* levels ≤10% at 3 months. The molecular responses at 3 months after specific TKI therapy are shown in Table 3.

**Table 3:** Molecular responses for each specific TKI at 3 months.

*BCR-ABL1* level at 3 months: % (n)	Imatinib	Nilotinib	Dasatinib	Bosutinib
**>10%**	9% (6/67)	0% (0/34)	0% (0/10)	0% (0/1)
**≤10%**	91% (61/67)	100% (34/34)	100% (10/10)	100% (1/1)
**Not evaluated (n)**	6	0	7	0

We observed that when using the Xpert *BCR-ABL1* assay, the 10% cutoff at 3 months was not associated with the probability of achieving CCyR (50% vs. 87%, p = 0.1) or MMR (60% vs. 80%, p = 0.29) by 12 months (Table 4). To identify a *BCR-ABL1* level correlated with the achievement of MMR and CCyR at 12 months, we used an ROC curve to identify the optimal cutoff that would facilitate the classification of patients as high or low risk with maximal sensitivity and specificity. The area under the curve (AUC) of the ROC curve was 0.88. At 3 months, patients with transcript levels below 1.5% showed significantly better probabilities of CCyR response at 12 months, with 81% and 94% sensitivity and specificity, respectively, for CCyR. With this new cutoff, the probabilities for CCyR and MMR at 12 months were 98% vs. 54% (p<0.001) and 88% vs. 56% (p<0.001), respectively (Table 5). These results were also corroborated in the group of patients treated exclusively with imatinib after excluding those treated with frontline 2GTKI (p = 0.003). The probability of treatment changes was also greater for patients with a molecular response > 1.5% at 3 months (42.9% vs. 16.9%, p = 0.005), and this cutoff also identified the probability of achieving MMR or stronger responses at any time (100% vs. 85%, p = 0.002).

**Table 4:** Probabilities of complete cytogenetic response and major molecular response at 12 months according to the molecular response (≤10%) at 3 months.

	CCyR (p = 0.1)		MMR (p = 0.29)	
	No	Yes	No	Yes
**>10%**	2 (50%)	2 (50%)	2 (40%)	3 (60%)
**≤10%**	14 (13%)	92 (87%)	20 (20%)	78 (80%)

**Table 5:** Probabilities of complete cytogenetic responses and major molecular responses at 12 months according to the molecular response (≤1.5%) at 3 months.

	CCyR (p = <0.001)		MMR (p = <0.001)	
	No	Yes	No	Yes
**>1.5%**	15 (46%)	18 (54%)	14 (44%)	18 (56%)
**≤1.5%**	1 (2%)	76 (98%)	8 (12%)	63 (88%)

Using MMR with a 12-month endpoint, we generated a multivariate regression model that included the variables listed in Table 1 together with the molecular response at 3 months (cutoff of >1.5). The effect of the molecular response at 3 months on MMR at 12 months was not affected through other variables (OR = 68.9; 95% confidence interval: 8.4–568.3, p<0.001).

### Validation of the 1.5% cutoff in an independent cohort of CML patients treated with frontline 2GTKI

Because most of the patients in the study cohort were treated with frontline imatinib, we validated the observed results in a cohort of CML patients treated with 2GTKI as first-line therapy. A total of 57 consecutive patients from *Andalusian CML Group Registry* were examined with a median follow-up of 38 months (3–56). All patients were monitored using the Xpert *BCR-ABL1* assay. The median age was 48 years (18–74). The ratio of men to women was 59/41, and the risk groups according to Sokal Score were 48%, 30% and 22% for low, intermediate and high risk, respectively. First-line treatment consisted of nilotinib and dasatinib in 58% and 42% of patients, respectively. Overall, the probability of achieving CCyR and MMR at 12 months was 92% (48/52) and 82% (39/47), respectively. Ten patients (17%) required treatment changes as a result of resistance (n = 3), not achieving MMR (n = 3) or intolerance (n = 4). No patients progressed to advanced phases, and only 1 patient died during follow-up (not CML related). The overall median value of *BCR-ABL1* at 3 months was 0.16%.

Consistent with the original cohort of patients treated with first-line 2GTKI, all patients achieved a *BCR-ABL1* level ≤10% at 3 months; therefore, this threshold did not predict further evolution of the disease. We classified this new cohort based on the new cutoff observed in the primary population (*BCR-ABL1* level at 3 months ≤1.5%); 77% of patients achieved *BCR-ABL1* levels ≤1.5%, whereas 23% of patients did not. This cutoff also predicted the probability to obtain MMR by 12 months (91% vs. 44% (p = 0.025).

## Discussion

The degree of molecular response before reaching CCyR was not relevant until some years ago, when several groups showed that a new threshold of 10% at 3 months identified a group of patients with different probabilities of achieving CCyR, MMR, and MR4.5 as well as PFS and OS [13–17]. Therefore, achieving a molecular response ≤10% at 3 months has been included in the guidelines as a new surrogate endpoint that should be reached to maximize favorable outcomes [3,19,20]. Nonetheless, this study is the first to examine the association between molecular responses at 3 months using the Xpert *BCR-ABL1* assay.

Automated molecular platforms, such as GeneXpert, are currently used in many centers as the only molecular procedure for monitoring CML patients. This increased use primarily reflects the simplicity and rapidity of the automated procedure compared with standardized RQ-PCR. As previously described, our studies and those of others have reported a good correlation between automated procedures and standardized RQ-PCR for monitoring patients once CCyR has been achieved. Regardless, a good correlation has not been observed in patients with *BCR-ABL1* levels >10% [8–12].

In our study, we showed how a 10% *BCR-ABL1* threshold at 3 months, measured using the Xpert *BCR-ABL1* method, does not predict CCyR or MMR. We propose that this discrepancy is due to the low number of patients with *BCR-ABL1* levels above 10% and likely reflects the overestimation of molecular responses in patients without CCyR using the Xpert assay. We observed that nearly 94% of the patients treated with imatinib obtained an optimal response (≤10%) at 3 months. Moreover, all patients treated with 2GTKI in the original and validation cohorts achieved *BCR-ABL1* levels of ≤10% at 3 months.

We determined a new cutoff using an ROC curve; this new cutoff of 1.5% predicted the probabilities of achieving MMR and CCyR at 12 months and also the probabilities for treatment changes and deep molecular responses when *BCR-ABL1* levels are measured using the Xpert method. Moreover, this new threshold was observed as the only factor for predicting responses at 12 months compared with classical risk factors, including sex, age and even the Sokal risk index.

The significantly lower rate of MMR at 12 moths for patients with *BCR-ABL1* values>1.5% at 3 months is consistent with the reports by Branford et al. [15] and Hughes et al. [18]. In these studies, the predictive threshold at 3 months, by RQ-PCR (IS), was found at 1.45% and 1%, respectively, values that are very similar to those obtained in the present study using the Xpert *BCR-ABL1* assay.

There were no differences in progressions or OS, likely reflecting the low number of events. In the cohort examined in the present study, most of the patients (58%) were treated with imatinib first-line therapy, and the results were similar when considering only this subgroup of patients.

Currently, a significant percentage of patients are treated with first-line 2GTKI; therefore, we validated the value of the new cutoff using the Xpert system in an independent cohort of patients treated with frontline 2GTKI therapy.

The main limitation of this study was the lack of simultaneous monitoring with a *BCR-ABL1* (IS) EUTOS method, which should be addressed in a future study.

In conclusion, we do not question the 10% cutoff at three months analyzed by the standard RQ-PCR method. However, we did show that when using the current version of Xpert *BCR-ABL1*, a cutoff of 1.5% at 3 months can better identify patients with different probabilities of achieving an optimal response at 12 months. This threshold was validated in an independent sample, and the finding provides a basis for future, larger studies of the use of this practical and widespread method.
